# The localization and lateralization of fear aura and its surgical prognostic value in patients with focal epilepsy

**DOI:** 10.1002/acn3.51607

**Published:** 2022-06-14

**Authors:** Qian Cao, Tao Cui, Qun Wang, Zhi‐Mei Li, Shang‐Hua Fan, Zhe‐Man Xiao, Song‐Qing Pan, Qin Zhou, Zu‐Neng Lu, Xiao‐Qiu Shao

**Affiliations:** ^1^ Department of Neurology Renmin Hospital of Wuhan University Wuhan 430060 China; ^2^ Department of Neurology Beijing Tiantan Hospital, Capital Medical University Beijing 100070 China; ^3^ China National Clinical Research Center for Neurological Diseases Beijing 100070 China; ^4^ Beijing Institute of Brain Disorders, Collaborative Innovation Center for Brain Disorders Capital Medical University Beijing 100070 China

## Abstract

**Objective:**

Fear aura has traditionally been considered relevant to epileptic discharges from mesial temporal areas, and few studies have investigated its effect on surgical outcome in drug‐resistant epilepsy. We aim to assess the localizing and lateralizing value as well as prognostic significance of fear aura in patients with focal epilepsy.

**Methods:**

The occurrence of fear aura in relation to epileptogenic origin and its association with postoperative outcome were analyzed in 146 consecutive patients undergoing resective surgery for intractable epilepsy.

**Results:**

Ninety‐four (64.4%) patients reported auras, and 31 (21.2%) reported fear aura in their seizures. One hundred ten (75.3%) patients had an Engel class I outcome until last follow‐up, of whom 24 experienced fear aura preoperatively. Fear aura appeared more frequently during temporal and frontal lobe seizures, but did not lateralize the seizure onset zone. There were no significant baseline differences between patients with and without fear aura. No correlation was found between postoperative outcome and the presence of auras. Occurrence of fear aura failed to show predictive value in surgical outcome whether in pooled or subgroup analysis.

**Interpretation:**

This study advances our understanding of the origin of fear aura, and is helpful for presurgical evaluation and outcome prediction. Without lateralizing value, fear aura is more commonly seen with temporal or frontal origin. When taken as a whole, auras do not have a significant impact on seizure outcome in focal epilepsy. Patients with fear aura are no more likely to become seizure‐free than those without fear aura.

## Introduction

Epilepsy surgery is a preferred option for patients with drug‐resistant focal epilepsy. Despite progress in presurgical evaluation of epilepsy, the success of epilepsy surgery has not remarkably improved over recent decades. Long‐term outcome studies suggest that the rates of seizure freedom 10 years postoperatively vary from 35% to 62%.^1^, ^2^, ^3^ Surgical prognosis largely relies on accurate presurgical localization of the epileptogenic zone, but it cannot be directly and definitively identified by clinical information or auxiliary examination indices. In this situation, multiple sources of clinical data should be fully utilized to define the presumptive epileptogenic zone and predict postsurgical outcome.

Auras, defined as subjective ictal symptoms that precede seizures, constitute a cardinal hallmark of focal epilepsy and may have localizing and in certain cases lateralizing value in determining the epileptogenic zone.^4^, ^5^ Fear is one of the affective symptoms and is common in mesiotemporal lobe epilepsy. However, although the role of temporal limbic structures, especially the amygdala, in the mediation of fearful emotion is well established,^6^, ^7^ the seizure origin of fear has also been found to originate from mesial frontal regions,^8^ occipital,^9^, ^10^ and parietal^11^ lobes; besides, there has been no consensus as to its lateralizing significance.^12^, ^13^, ^14^ In addition, most evidence on the localization of fear comes from studies of electrical stimulation,^15^, ^16^, ^17^ or studies of scalp or intracranial electroencephalography (EEG) recordings,^8^, ^18^ while comparably few studies take elimination or significant reduction in seizure frequency following surgical resection as verification of originally presumed localization, which determines seizure onset beyond reasonable doubt and is closer to actual clinical practice. Therefore, these problems merit re‐examination.

Reliable predictors for seizure outcome are important to ensure prompt referral of appropriate candidates to presurgical evaluation and epilepsy surgery. Being the initial symptom of epileptic seizure and readily available clinical data, auras should be given more attention. However, to our knowledge, only two studies to date has directly addressed the relationship of fear aura to seizure outcome following epilepsy surgery, but unfortunately with inconsistent results.^14^, ^19^ Moreover, the two studies concerned the prognostic role of fear aura in temporal lobe epilepsy (TLE) rather than in a broader spectrum of focal epilepsy. Thus, further investigation is necessary to clarify these issues.

Herein, we analyzed the clinical data of a cohort of patients undergoing resective epilepsy surgery to systematically assess the localization and lateralization of fear aura, and to particularly investigate the possible correlation, if any, between the presence of fear aura and postsurgical seizure outcome.

## Methods

### Patient selection

This was a retrospective observational study of a prospectively acquired database. One hundred fifty consecutive patients with intractable epilepsy underwent resective epilepsy surgery at the tertiary epilepsy center of Tiantan Hospital from January 2015 to October 2020. All their clinical details archived were reviewed. There were no restrictions on age or gender to enter the study. The diagnosis of medically refractory focal epilepsy was made by three experienced epileptologists (Q. Wang, Z.‐M. Li, and X.‐Q. Shao) in this epilepsy center according to the International League Against Epilepsy (ILAE) definition.^20^ Patients were selected for surgery based on a comprehensive presurgical evaluation and a detailed discussion in the multidisciplinary epilepsy conference. The presurgical evaluation included clinical examination, 3.0 Tesla cranial magnetic resonance imaging (MRI), long‐term scalp video EEG monitoring, positron emission tomography (PET), and intracranial EEG when deemed necessary. Patients were followed up at 3 and 12 months postoperatively and at least once a year thereafter. Patients unable to attend a direct outpatient or inpatient visit were contacted by telephone interview to complete a semi‐structured questionnaire for seizure recurrence if any and its date of recurrence, and status of current anti‐seizure medications (ASMs). Outcome data were collected directly from each patient and witnesses whenever possible. Patients with a minimum of 1 year follow‐up were included in this study.

This study was approved by the ethics committee of Beijing Tiantan Hospital and was performed according to the Declaration of Helsinki. All patients gave informed consent for participation and written consent to permit the publication of clinical data.

### Assessment of postoperative outcome, identification of fear aura, and definition of lateralization and localization of epileptic focus

All outcome data were submitted to three epileptologists (Z.‐M. Xiao, S.‐Q. Pan, and Q. Zhou) blinded to the study for assessment of postoperative seizure outcome according to the Engel's classification.^21^ When there was uncertainty in the process of assessment, consensus was reached through discussion. Favorable outcome corresponded to Engel class I after surgery until the last follow‐up, irrespectively of the status of ASMs, whereas others achieving Engel class II–IV outcomes were considered as having an unfavorable outcome.

Information on fear aura was obtained through review of medical record database. The fear aura was referred to a sudden, often brief, fearful affect at the beginning of an epileptic seizure, which occurred without context and was not the fear of an impending seizure; the fear aura was deemed present if related to at least one, but not necessarily all, of the patient's seizures. The lateralization and localization of epileptic focus were defined by the localization of surgical resection sites and determined only in those patients who had favorable outcomes.

### Statistical analysis

Continuous variables with skewed distribution were presented as median and range, and those with normal distribution were presented as mean and standard deviation (SD). Categorical variables were expressed as counts and percentages. Pearson's chi‐squared test or Fisher's exact test in case of small subgroup sample size was used for categorical variables. Continuous variables between two independent groups were compared using Student's *t*‐test or nonparametric Mann–Whitney *U* test where appropriate. Kaplan–Meier survival analysis by log‐rank test was used to compare the probability of maintaining Engel class I outcome throughout the follow‐up between groups. Kaplan–Meier survival curves were plotted using GraphPad Prism 7.0. Statistical analyses were performed using SPSS 20.0 and *p* value <0.05 was considered statistically significant.

## Results

### Cohort characteristics

**Figure 1:**
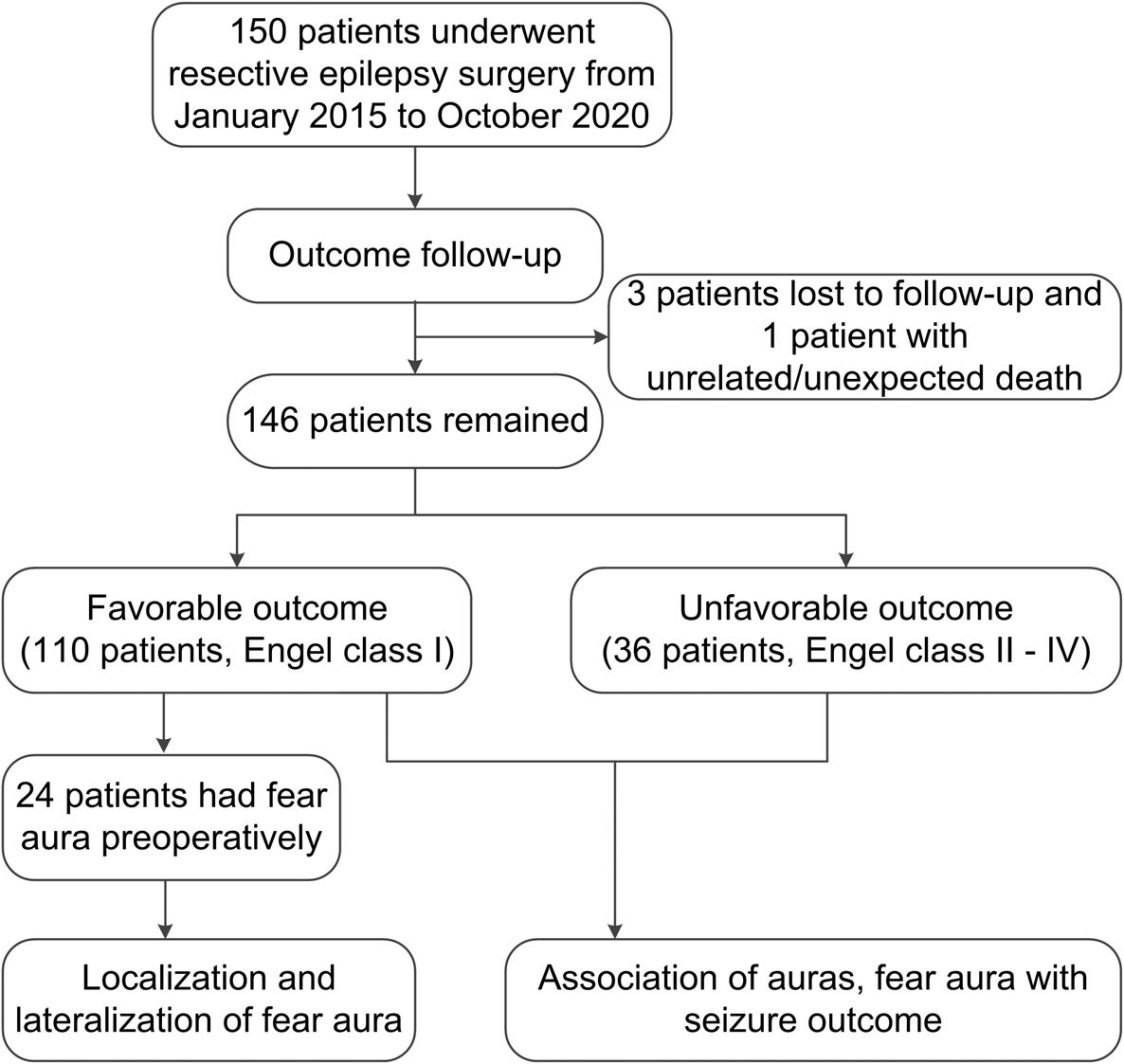
Flow chart showing the study protocol.

The study design is depicted in Figure 1. Three patients were lost to follow‐up at 16, 26, and 33 months after surgery, respectively; one patient died from car accident 55 months after surgery. The final cohort, therefore, included 146 patients (85 males and 61 females). The mean age at seizure onset was 13.0 ± 8.6 years (range 0–36 years), the mean duration of epilepsy prior to surgery 13.9 ± 9.4 years (range 1–41 years), and the mean age at surgery 26.9 ± 9.7 years (range 9–56 years). Past history of initial precipitating injury (IPI) was noted in 85 (58.2%) patients: febrile seizures in 25 (17.1%), perinatal insult in 19 (13.0%), meningitis/encephalitis in 11 (7.5%), head trauma with loss of consciousness (LOC) in 5 (3.4%), and head trauma without LOC in 25 (17.1%).

Ninety‐four (64.4%) patients reported auras, and 31 (21.2%) had fear aura. Thirty‐five (24.0%) cases had focal aware seizure (FAS), 124 (84.9%) had focal impaired awareness seizure (FIAS), and 116 (79.5%) had focal to bilateral tonic–clonic seizure (FBTCS). One hundred eighteen (80.8%) patients had lesional MRI, and 63 (43.2%) required invasive EEG evaluation.

Sixty‐nine (47.3%) patients underwent left‐sided surgery. Ninety‐three (63.7%) patients had temporal lobe surgery, 19 (13.0%) had frontal lobe surgery, eight (5.5%) had parietal lobe surgery, six (4.1%) had occipital lobe surgery, four (2.7%) had insular lobe surgery, and 16 (11.0%) had multilobar surgery. Seventy‐one (48.6%) patients underwent anterior temporal lobectomy (ATL), namely, anterior two‐thirds temporal lobectomy combined with amygdalohippocampectomy (AHE), 60 (41.1%) underwent lesionectomy, four (2.7%) underwent selective AHE, three (2.1%) underwent AHE with lesionectomy, and eight (5.5%) underwent multilobar resection. Histopathologic examinations revealed hippocampal sclerosis in 54 (37.0%) patients, cortical dysplasia in 44 (30.1%), ganglioglioma in 9 (6.2%), dysembryoplastic neuroepithelial tumor in four (2.7%), other benign brain tumors (astrocytoma, papillary glioneuronal tumor) in five (3.4%), vascular malformation in nine (6.2%), and other pathology (e.g., gliosis, encephalomalacia, non‐specific, and brain damage) in 21 (14.4%).

### Postoperative outcome

The cohort was followed up for a median duration of 31 months (range 12–67 months). One hundred ten (75.3%) patients remained free of disabling seizures (Engel class I) until their last follow‐up, while 11 (7.5%) had rare disabling seizures (Engel class II), 10 (6.8%) had a reduction in seizure frequency of greater than 80% (Engel class III), and 15 (10.3%) reported no worthwhile improvement (Engel class IV).

### Localization and lateralization of fear aura

Of the 110 patients who had favorable outcomes, 24 experienced fear aura preoperatively and had become seizure‐free without auras (Engel class IA), among whom 15 (62.5%) had TLE, seven (29.2%) had frontal lobe epilepsy (FLE) and two (8.3%) had multilobar epilepsy (MLE). Hippocampal sclerosis (10, 41.7%) and cortical dysplasia (10, 41.7%) were the two most common pathological types in these 24 patients. The distribution of fear aura in different types of focal epilepsy is shown in Table 1. The remaining seven patients with fear aura had unfavorable outcomes: two had an Engel class II outcome, two had an Engel class III outcome, and three had an Engel class IV outcome. Table S1 demonstrates the demographic, clinicopathologic, radiologic, electrophysiological, and surgical characteristics of these 31 patients.

**Table 1:** Distribution of fear aura in different types of focal epilepsy.

	TLE (*n* = 69)	FLE (*n* = 14)	PLE (*n* = 7)	OLE (*n* = 5)	ILE (*n* = 3)	MLE (*n* = 12)	*N* = 110^a^
Fear aura *n* = 24 (%)	15 (62.5)	7 (29.2)	0 (0)	0 (0)	0 (0)	2^b^ (8.3)	

TLE, temporal lobe epilepsy; FLE, frontal lobe epilepsy; PLE, parietal lobe epilepsy; OLE, occipital lobe epilepsy; ILE, insular lobe epilepsy; MLE, multilobar epilepsy.

^a^ Only the 110 patients were included who had Engel class I outcome.

^b^ One was parieto‐occipital junction epilepsy and the other was temporo‐occipital junction epilepsy.

Because of the predominant distribution of fear aura in TLE and FLE, we compared the localizing value of fear aura between patients with TLE/FLE and those with epileptic focus outside the temporal and frontal lobes (extra‐TLE/FLE). TLE/FLE patients were characterized by a higher proportion of fear aura compared to extra‐TLE/FLE patients (*p* = 0.037); the fear aura appeared to be more common in FLE, but the differences did not reach statistical significance (*p* = 0.064); as for lateralization of fear aura, there were no significant differences between left‐sided and right‐sided epilepsies (*p* = 0.534) (Table 2).

**Table 2:** Localization and lateralization values of fear aura in focal epilepsy.

	Localization value						Lateralization value		
	TLE + FLE *n* = 83 (%)	Ex‐(TLE + FLE) *n* = 27 (%)	*p* value	TLE *n* = 69 (%)	FLE *n* = 14 (%)	*p* value	Left *n* = 52 (%)	Right *n* = 58 (%)	*p* value
Fear aura present	22 (26.5)	2 (7.4)	**0.037**	15 (21.7)	7 (50.0)	0.064	10 (19.2)	14 (24.1)	0.534
Fear aura absent	61 (73.5)	25 (92.6)		54 (78.3)	7 (50.0)		42 (80.8)	44 (75.9)	

Bold type indicates significance. Only the 110 patients were included who had Engel class I outcome.

TLE, temporal lobe epilepsy; FLE, frontal lobe epilepsy; Ex‐(TLE + FLE), extratemporal and extrafrontal lobe epilepsy.

### Association between auras, fear aura, and seizure outcome

We did not find a significant association between auras and postoperative outcome (*p* = 0.943) (Table 3). Next, in order to have relatively comparable groups for the purpose of validating whether fear aura was an independent prognostic variable predicting seizure outcome, we compared the baseline characteristics of the patients presenting with and without fear aura (Table 4). There were no significant differences between the two groups in terms of gender, past history of IPI, seizure type, presence of MRI lesion, surgical approach, and pathological type. Age at onset, age at surgery, duration of epilepsy, and duration of follow‐up did not differ between the two groups either. As depicted in Table 5, the presence of fear aura did not correlate significantly with seizure outcome (*p* = 0.762). We then did a subgroup analysis based on surgical site to ascertain if the association of fear aura with seizure outcome was affected by resection site of epilepsy surgery; however, the presence of fear aura was still not significantly different between the favorable and unfavorable outcome groups in both temporal lobe (*p* = 0.742) and frontal lobe (*p* = 0.338) surgery subgroups.

**Table 3:** Association of auras with seizure outcome in the whole cohort.

	Favorable outcome *n* = 110 (%)	Unfavorable outcome *n* = 36 (%)	*p* value
Auras present	71 (64.5)	23 (63.9)	0.943
Auras absent	39 (35.5)	13 (36.1)	

**Table 4:** Demographic and clinical characteristics of patients with and without fear aura.

	With fear aura *n* = 31 (%)	Without fear aura *n* = 115 (%)	*p* value
Duration of follow‐up (months): mean ± SD/median (range)	37.2 ± 16.4	30 (12–67)	0.256^b^
Female	16 (51.6)	45 (39.1)	0.211
Mean age at onset (years) ± SD	12.3 ± 7.5	13.2 ± 8.9	0.597
Mean duration of epilepsy (years) ± SD	15.9 ± 9.3	13.4 ± 9.3	0.173
Mean age at surgery (years) ± SD	28.2 ± 10.5	26.5 ± 9.5	0.387
Past medical history			
Febrile seizures	7 (22.6)	18 (15.7)	0.363
Perinatal insult	3 (9.7)	16 (13.9)	0.748
Meningitis/encephalitis	3 (9.7)	8 (7.0)	0.900
Head trauma with LOC	0 (0)	5 (4.3)	0.532
Head trauma without LOC	4 (12.9)	21 (18.3)	0.482
Seizure type^a^			
FAS	9 (29.0)	26 (22.6)	0.457
FIAS	27 (87.1)	97 (84.3)	0.923
FBTCS	23 (74.2)	93 (80.9)	0.414
MRI lesional	26 (83.9)	92 (80.0)	0.627
Type of surgery			
ATL	18 (58.1)	53 (46.1)	0.236
Lesionectomy	10 (32.3)	50 (43.5)	0.260
AHE	1 (3.2)	3 (2.6)	1.000
AHE + lesionectomy	0 (0)	3 (2.6)	1.000
Multilobar resection	2 (6.5)	6 (5.2)	1.000
Pathology			
Hippocampal sclerosis	14 (45.2)	40 (34.8)	0.288
Cortical dysplasia	11 (35.5)	33 (28.7)	0.465
Ganglioglioma	1 (3.2)	8 (7.0)	0.729
DNET	1 (3.2)	3 (2.6)	1.000
Other benign brain tumors	1 (3.2)	4 (3.5)	1.000
Vascular malformation	1 (3.2)	8 (7.0)	0.729
Other (e.g., gliosis, encephalomalacia, brain damage, non‐specific)	2 (6.5)	19 (16.5)	0.259

LOC, loss of consciousness; FAS, focal aware seizure; FIAS, focal impaired awareness seizure; FBTCS, focal to bilateral tonic–clonic seizure; ATL, anterior temporal lobectomy; AHE, amygdalohippocampectomy; DNET, dysembryoplastic neuroepithelial tumor.

^a^ The seizure type percentages did not add up to 100% because some patients had more than one type of seizure.

^b^ Mann–Whitney *U* test was used to compare follow‐up duration, and other continuous variables were compared by Student's *t*‐test.

**Table 5:** Association of fear aura with seizure outcome in the whole cohort, in those with temporal lobe surgery and in those with frontal lobe surgery.

	Whole cohort		Temporal lobe surgery		Frontal lobe surgery	
	*N*	Fear aura *n* (%)	*N*	Fear aura *n* (%)	*N*	Fear aura *n* (%)
Favorable outcome	110	24 (21.8)	69	15 (21.7)	14	7 (50.0)
Unfavorable outcome	36	7 (19.4)	24	6 (25.0)	5	1 (20.0)
*p* value		0.762		0.742		0.338

**Figure 2:**
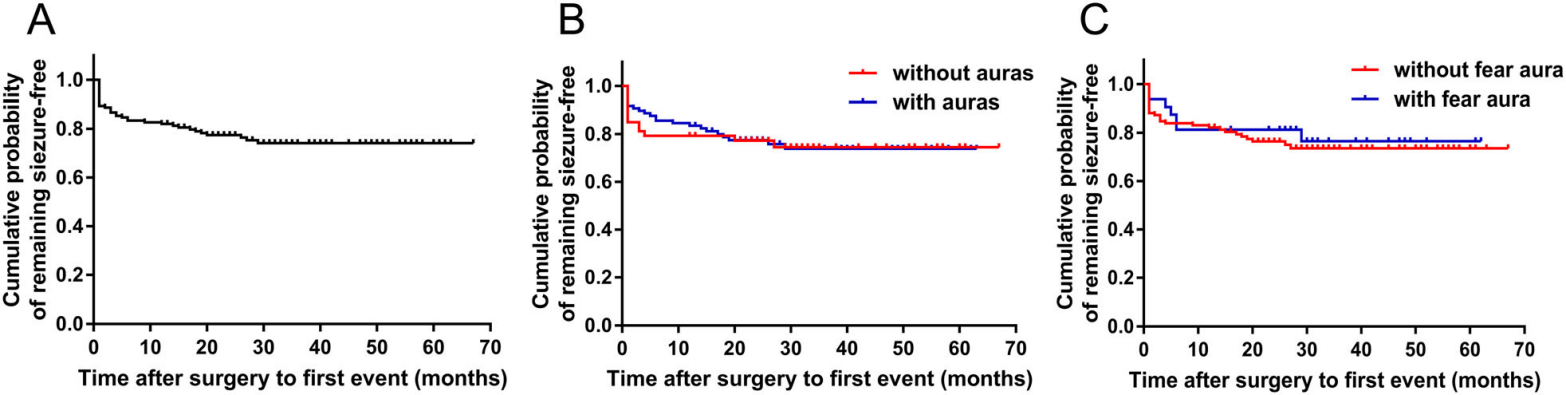
Kaplan–Meier survival curves for seizure recurrence after epilepsy surgery in the whole cohort (*n* = 150^a^). (A) The Kaplan–Meier survival curve presented the probability that patients will maintain a favorable outcome over the follow‐up period. (B) Postoperative seizure outcome was not significantly different in patients with auras (blue line) compared with those without auras (red line) (*p* = 0.919, log rank test). (C) Probability of seizure freedom was similar in patients with fear aura (blue line) to those without fear aura (red line) (*p* = 0.700, log rank test). ^a^The cohort in survival analysis also included the three patients lost to follow‐up and the patient with unrelated/unexpected death, besides those whose postoperative evaluation was available until the last follow‐up. [Colour figure can be viewed at wileyonlinelibrary.com]

The Kaplan–Meier survival analysis for the whole cohort is shown in Figure 2A. The chance of seizure freedom was about 82% by 1 year. Beyond this, the rate of drop in chance of seizure‐free status was relatively slow from 82% to 74% over the next 17 months after which the curve tended to be flat for the rest of the follow‐up period. The survival curves for patients with or without auras maintaining Engel class I outcome throughout the follow‐up period lacked significant differences (*p* = 0.919) (Fig. 2B). The results of Kaplan–Meier analysis did not show a direct correlation between presence of fear aura and postoperative seizure freedom (*p* = 0.700) (Fig. 2C).

## Discussion

In the current study, we demonstrated that occurrence of fear aura was associated with temporal or frontal seizure focus, but not with laterality of seizure focus. Reporting of fear aura was not predictive of postsurgical seizure outcome.

### Prevalence of fear aura

The prevalence of fear aura in patients with focal epilepsies is variable ranging from 7% to 36%.^13^, ^14^, ^19^, ^22^ This discrepancy probably reflects methodologic differences in patient selection and heterogeneities of etiology. Chong et al. selected patients with focal or localization‐related epilepsy from the Epilepsy Phenome/Genome Project (EPGP) database which only included epilepsy subjects of unknown cause, and reported a prevalence of fear aura to be 7%^13^; on the other hand, Feichtinger et al. found a relatively high prevalence of 36%, because the study was limited to TLE with mesial temporal sclerosis and the frequency of fear could conceivably be higher given the pivotal role of mesial temporal regions in the expression of fear.^19^ Our cohort comprised a heterogeneous group of patients with different epileptic foci of variable etiology, and 21.2% of the patients reported fear aura, which fell between the results of the above‐mentioned two studies.

### Localizing value of fear aura

Our study revealed that fear aura was most often localized to the temporal and frontal lobe. These results are consistent with previous studies demonstrating that fear is among the most common experiential phenomenons elicited by temporal lobe epileptic discharge.^17^, ^23^ Adding to this, we found that the frontal lobe also played a pivotal role in the formation of fearful emotion because half of the patients who had FLE with favorable outcome had fear aura. This proportion appeared even higher than that in patients with TLE (50.0% vs. 21.7%), although the difference was not statistically significant. The favored locations of fear aura in temporal and frontal lobes resonate the work of Biraben et al. who have argued that the network of ictal fear involves orbitoprefrontal, anterior cingulate, and temporal limbic cortices.^8^ Also noteworthy is that two patients in our study had fear aura of parieto‐ and temporo‐occipital origins, respectively. Similar situations, albeit rarely, has been described in earlier studies,^9^, ^10^, ^11^ which reported ictal fear as leading symptom originating from the occipital or parietal lobe. The mechanisms are proposed to be the secondary propagation of discharges to the symptomatogenic zone in the amygdala, or the early emotional network independent of amygdala activation, in which case the occipital and parietal cortex are also involved in fear processing.

### Lateralizing significance of fear aura

The question of the lateralizing value of fear aura has long been puzzling. Glascher et al. proposed that, while both amygdalas are specialized for fear, the right one is an initial, brief and automatic fear processor, whereas the left one appears to be more discriminated and detail‐oriented.^24^ Accordingly, early ictal fear, namely fear aura, should occur more often from the right temporal lobe epileptic focus. Such a mechanism is consistent with the findings of Chong et al., who found that the right hemispheric laterality of seizure was statistically more common in patients reporting fear.^13^ Additional support for this model comes from the trend toward right epileptic focus causing fear aura (68% right vs. 32% left) in a review of 144 published cases.^12^ However, the work of Palmini et al., Chiesa et al., and Toth et al. failed to confirm a clear association between fear aura and cerebral lateralization.^14^, ^18^, ^22^ Furthermore, a systematic research exclusively involving children reported that negative emotions did not lateralize the seizure onset zone.^25^ Our study also did not find a lateralizing significance for fear aura in focal epilepsy, although there was a slight preponderance of right‐sided laterality (24.1% right vs. 19.2% left). The uncertainty of laterality of fear aura might be explained by the fact that clinical manifestations of fear aura cannot be simply confined to an absolutely single hemisphere, as fear is a multifaceted process that needs communication across brain regions and involvement of complex circuits.^26^, ^27^ Another point deserving attention is the different fear intensities manifested during an epileptic seizure: the intensity may vary from a slight trace of insecurity to an intense feeling of horror, leading to varying levels of participation of two hemispheres; for instance, the right hemisphere is enough to produce mild fear, but intense fear entails co‐operations of two hemispheres. This assumption necessitates a deeper questioning into patients' reports of fear aura in medical history collection. In view of these ambiguities, fear aura cannot currently be utilized as lateralizing sign in everyday clinical practice.

### Prognostic value of auras and fear aura in epilepsy surgery

Our study did not find prognostic value for auras in predicting postsurgical outcome. The result corroborates many previous studies,^28^, ^29^, ^30^, ^31^ except for those of Sperling et al.^32^ and Arifin et al.^33^ The former argued for favorable prognostic significance of auras for patients undergoing temporal lobectomy; however, strictly speaking, their finding did not reach statistical significance (*p* = 0.06), although the trend was toward a greater likelihood of being free of seizure in patients with auras than in those without auras. Similarly, the latter demonstrated occurrence of auras conveyed a favorable prognosis in TLE patients, but a baseline assessment among the two groups was not attempted, which may introduce potential confounding factors. Actually, most auras have their own anatomical localization, so it is more appropriate to evaluate the respective roles of aura subtypes in certain types of focal epilepsy rather than to treat auras as a whole. For example, patients with mesial TLE who reported abdominal auras had a better postoperative prognosis regarding seizure control compared with those who did not^29^; conversely, presence of auras historically associated with extratemporal structures (e.g., somatosensory, visual, and dysphasic auras) or lateral temporal structures (e.g., auditory and vertiginous auras) in mesial TLE patients may herald a more widespread epileptogenic zone, and thereby a lower chance of seizure control after surgery.^30^, ^34^, ^35^, ^36^ Therefore, aura subtypes may be associated with seizure outcome in some circumstances, but auras, when analyzed as a whole, do not have a significant bearing on seizure outcome.

Given that fear aura most often denotes the presence of a temporal or frontal lobe epileptic focus, we hypothesized that patients with fear aura would have a better prognosis; however, this is not necessarily the case. The current study observed no significant correlation between fear aura and seizure outcome. Our results were in line with the study of Toth et al.,^14^ but Feichtinger et al.^19^ demonstrated evidence to the contrary which found fear aura is a positive predictor for postoperative seizure control. Regarding this controversy, there are three aspects that need to be illustrated. First, the relative small sample size of 33 patients in the study of Feichtinger et al.^19^ may have produced subject selection bias; perhaps an analysis including more cases might increase the statistical power and make the results more plausible. Second, the heterogeneity of disease and difference in surgical approach between studies partially contribute to the discrepancy of the results. Feichtinger et al.^19^ selected TLE patients with mesial temporal sclerosis who received anteromesial temporal resection; Toth et al.^14^ included mesial TLE or neocortical TLE patients of various etiologies who underwent ATL, AHE, or lesionectomy; our cohort contained a broader disease spectrum receiving resective epilepsy surgery. Considering these interference factors, we compared the baseline characteristics of the patients with and without fear aura, and found no significant differences; furthermore, we conducted subgroup analysis of predictive value of fear aura in patients with temporal and frontal lobe surgery, and this type of analysis, to our knowledge, has never been done before. Finally, the negative value of fear aura for seizure outcome may also be associated with the possibility that fear aura, at least in some cases, corresponds to seizure propagation rather than primary epileptogenic cortex, which is a problem that must be faced in all studies of seizure semiology; on this ground, the fear aura might be a false localizing sign. Consequently, despite definitive localizing meanings, the significance of fear aura in predicting seizure outcome is limited.

### Implications and limitations of the study

The clinical implications of the current study pertain both to presurgical evaluation and outcome expectation after surgery. Albeit without lateralizing value, the presence of fear aura favors involvement of temporal or frontal lobe, and therefore provides valuable information about the location of epileptogenic zone. However, the analysis of fear aura does not help to identify ideal candidates for a good operative outcome, so whether there is presence of fear aura should not serve as a barrier to epilepsy surgery for the patients who have concordant data on epileptogenic zone from objective clinical signs, EEG monitoring and structural imaging.

This study is also liable to some limitations. First, as a retrospective study of a prospectively acquired database that was not intended to focus on fear aura, we inevitably encountered difficulty in obtaining detailed descriptions and characteristics of fear aura (e.g., fear‐related emotional, cognitive, and autonomous signs; intensity of fear from a slight trace of insecurity to an intense feeling of horror), and thus, a direct and systematic questioning combined with corresponding video‐recorded ictal affective behavior are warranted to further explore this issue. Second, the follow‐up time in this study is at least 1 year, which presumably is not long enough for the occurrence of cases with unfavorable outcome; the relatively low frequency of unfavorable outcome might have underpowered our study and led to negative results. Third, we must acknowledge that the heterogeneity of pathology and difference in surgical modality might render our results less reliable to some extent, although we compared the baseline characteristics and did subgroup analysis; therefore, a multicenter prospective study with a much larger homogeneous patient cohort receiving a certain surgical approach should be done to validate our conclusion.

## Conclusion

This study strengthens our understanding of the localizing and lateralizing value of fear aura and is helpful in presurgical evaluation: without lateralizing significance, fear aura correlates with temporal or frontal lobe onset on most occasions. Our study is also instructive in outcome expectation and patient counseling: when taken as a whole, auras do not have a significant impact on seizure outcome; as for a certain aura subtype, patients with fear aura are no more likely to become seizure‐free than those without fear aura.

## Author Contributions

Cao and Cui contributed to the study concept and design. Cao drafted the manuscript. Shao supervised the study and critically revised the manuscript for important intellectual content. Cao and Fan collected and collated data. Xiao, Pan, and Zhou analyzed the outcome data and assessed the postoperative seizure outcome. Lu provided statistical expertise. Wang and Li provided administrative, technical, and material support.

## Conflict of Interest

On behalf of all authors, the corresponding author confirms no conflict of interest.

## Supporting information


**Table S1** Clinical features, MRI findings, seizure onsets, surgical types, pathological data, and outcomes of the 31 patients with fear aura.
