# Supplementary material for: The localization and lateralization of fear aura and its surgical prognostic value in patients with focal epilepsy
Source: Ann Clin Transl Neurol. 2022 Jun 14;9(8):1116–24. doi: 10.1002/acn3.51607 (PMC9380142; doi:10.1002/acn3.51607)
Supplement: Supplementary file 1 — Table S1 Clinical features, MRI findings, seizure onsets, surgical types, pathological data, and outcomes of the 31 patients with fear aura. [file ACN3-9-1116-s001.docx]

| **Supplementary table** Clinical features, MRI findings, seizure onsets, surgical types, pathological data and outcomes of the 31 patients with fear aura. | | | | | | | | | | |
| --- | --- | --- | --- | --- | --- | --- | --- | --- | --- | --- |
| **No** | **Sex** | **Age at onset** | **Duration of epilepsy** | **Seizure types** | **MRI lesions** | **EEG-monitoring** | **Seizure onset** | **Surgery** | **Pathology** | **Outcome**  **(Engel class)** |
| 1 | F | 18 | 17 | FIAS | Lt hippocampal abnormal signal | Scalp EEG | Lt temporal | ATL | HS | IA |
| 2 | F | 14 | 30 | FAS, FIAS, FBTCS | Lt HS | Scalp EEG | Lt temporal | ATL | HS | IA |
| 3 | F | 2 | 28 | FAS, FIAS, FBTCS | Lt parietal abnormal signal and atrophy | Scalp EEG | Lt parieto-occipital | Multilobar resection | Vascular malformation | IA |
| 4 | M | 12 | 5 | FIAS | Rt HS | Scalp EEG | Rt temporal | ATL | HS | IA |
| 5 | F | 14 | 31 | FAS, FBTCS | Abnormal signal in the Rt inferior frontal gyrus | Scalp EEG | Rt frontal | Lesionectomy | Ganglioglioma | IA |
| 6 | M | 12 | 30 | FIAS, FBTCS | Rt hippocampal abnormal signal | Scalp EEG | Rt temporal | ATL | HS | IA |
| 7 | M | 9 | 10 | FIAS | Rt HS | Scalp EEG | Rt temporal | ATL | HS | IA |
| 8 | M | 10 | 4 | FIAS, FBTCS | Normal (hypometabolism in the posterior segment of the Lt superior temporal sulcus shown by PET/MRI fusion) | Scalp EEG  Intracranial EEG | Lt temporal | Lesionectomy | Cortical dysplasia | IA |
| 9 | M | 1 | 24 | FIAS | Rt hippocampal atrophy | Scalp EEG | Rt temporal | ATL | HS | IA |
| 10 | M | 12 | 1 | FIAS, FBTCS | Abnormal signal in the Rt superior frontal gyrus | Scalp EEG  Intracranial EEG | Rt frontal | Lesionectomy | Cortical dysplasia | IA |
| 11 | M | 8 | 1 | FIAS | Rt anterior temporal abnormal signal | Scalp EEG | Rt temporal | ATL | Cortical dysplasia | IA |
| 12 | M | 14 | 12 | FIAS, FBTCS | Lt temporal softening lesion | Scalp EEG  Intracranial EEG | Lt temporal | Lesionectomy | Non-specific (neuronal loss, gliosis) | IA |
| 13 | M | 9 | 20 | FAS, FBTCS | Lt centrum semiovale abnormal signal | Scalp EEG | Lt frontal | Lesionectomy | Cortical dysplasia | IA |
| 14 | F | 17 | 24 | FIAS, FBTCS | Slightly full gyrus and thick cortex in Rt temporal-occipital  junction | Scalp EEG  Intracranial EEG | Rt temporal-occipital | Multilobar resection | Cortical dysplasia | IA |
| **Supplementary table** *（continued）* | | | | | | | | | | |
| **No** | **Sex** | **Age at onset** | **Duration of epilepsy** | **Seizure types** | **MRI lesions** | **EEG-monitoring** | **Seizure onset** | **Surgery** | **Pathology** | **Outcome**  **(Engel class)** |
| 15 | M | 1 | 22 | FIAS, FBTCS | Rt HS | Scalp EEG | Rt temporal | ATL | HS | IA |
| 16 | F | 22 | 8 | FIAS, FBTCS | Rt hippocampal abnormal signal and atrophy | Scalp EEG  Intracranial EEG | Rt frontal | Lesionectomy | Cortical dysplasia | IA |
| 17 | F | 3 | 22 | FIAS, FBTCS | Lt HS | Scalp EEG | Lt temporal | ATL | HS | IA |
| 18 | M | 21 | 3 | FIAS, FBTCS | Lt HS | Scalp EEG | Lt temporal | ATL | HS | IA |
| 19 | M | 29 | 7 | FIAS | Normal (hypometabolism in the Rt anterior temporal lobe and hippocampus shown by PET/MRI fusion) | Scalp EEG | Rt temporal | ATL | Cortical dysplasia | IA |
| 20 | M | 26 | 12 | FIAS, FBTCS | Rt mesiotemporal abnormal signal | Scalp EEG | Rt temporal | ATL | Astrocytoma | IA |
| 21 | M | 7 | 14 | FIAS, FBTCS | Normal (hypometabolism in the Rt anterior temporal lobe and hippocampus shown by PET/MRI fusion) | Scalp EEG  Intracranial EEG | Rt temporal | ATL | HS | IA |
| 22 | F | 12 | 20 | FAS, FIAS, FBTCS | Abnormal signal in the Rt inferior frontal gyrus | Scalp EEG | Rt frontal | Lesionectomy | Cortical dysplasia | IA |
| 23 | F | 17 | 13 | FAS, FBTCS | Bilateral frontal subcortical abnormal signal | Scalp EEG  Intracranial EEG | Lt frontal | Lesionectomy | Cortical dysplasia | IA |
| 24 | M | 3 | 16 | FIAS | Lt frontal abnormal signal | Scalp EEG  Intracranial EEG | Lt frontal | Lesionectomy | Cortical dysplasia | IA |
| 25 | F | 12 | 12 | FIAS, FBTCS | Abnormal signal in the Lt temporal pole | Scalp EEG  Intracranial EEG | Lt temporal | ATL | Cortical dysplasia | IIA |
| 26 | F | 8 | 25 | FIAS, FBTCS | Lt hippocampal atrophy | Scalp EEG | Lt temporal | ATL | HS | IIA |
| 27 | F | 8 | 16 | FAS, FIAS, FBTCS | Normal (hypometabolism in the Rt temporal lobe and hippocampus shown by PET/MRI fusion) | Scalp EEG | Rt temporal | ATL | HS | IIIA |
| **Supplementary table** *（continued）* | | | | | | | | | | |
| **No** | **Sex** | **Age at onset** | **Duration of epilepsy** | **Seizure types** | **MRI lesions** | **EEG-monitoring** | **Seizure onset** | **Surgery** | **Pathology** | **Outcome**  **(Engel class)** |
| 28 | F | 26 | 30 | FIAS | Rt mesiotemporal abnormal signal | Scalp EEG | Rt temporal | AHE | HS | IIIA |
| 29 | F | 8 | 22 | FBTCS | Lt HS | Scalp EEG | Lt temporal | ATL | HS | IVB |
| 30 | F | 19 | 5 | FAS, FIAS, FBTCS | Cystic lesion in the right cingulate gyrus | Scalp EEG | Rt frontal | Lesionectomy | DNET | IVA |
| 31 | F | 7 | 10 | FAS, FIAS, FBTCS | Bilateral hippocampal abnormal signal | Scalp EEG  Intracranial EEG | Rt temporal | ATL | Non-specific (neuronal loss, gliosis) | IVB |
| FIAS, focal impaired awareness seizure; FAS, focal aware seizure; FBTCS, focal to bilateral tonic-clonic seizure; Lt, left; Rt, right; HS, hippocampal sclerosis; DNET, dysembryoplastic neuroepithelial tumour; ATL, anterior temporal lobectomy; AHE, amygdalohippocampectomy. | | | | | | | | | | |
